# Book Reviews and Notices

**Published:** 1888-12

**Authors:** 


### BOOK REVIEWS AND NOTICES.

THE EAR AND ITS DISEASES; being Practical Contributions to the study of Otology. By SAMUEL SEXTON, M. D. Edited by CHRISTOPHER J. COLLES, M. D. With numerous original illustrations. New York: William Wood & Co. Chicago: W. T Keener. Pp. xii and 461. 1888.

In this work on the ear, the author has not attempted to cover the whole field of otology, but has selected certain subjects which he considered deserving of special attention. Some of them have been more or less fully treated of in other works upon the ear; others that are related directly or indirectly to the subject of otology are here presented in a way calculated to attract more attention to them than they have before received. The book reflects the ideas of the author based largely on his individual practice, which has been large and afforded him good opportunity of observing, and contributing to the growth of scientific otology, which is of so recent origin. Much as the author has restricted the scope of his work, it yet gives a good idea of the recent advances made in this specialty, and suggests how others can and should be made in the proper development of the subject. It thus becomes a valuable contribution to its literature.

A TEXT-BOOK OF HUMAN PHYSIOLOGY. By AUSTIN FLINT, M. D., LL. D., Professor of Physiology and Physiological Anatomy in The Bellevue Hospital Medical College, New York., etc., etc., etc. With three hundred and sixteen figures in the text, and two plates. Fourth edition, entirely rewritten. New York : D. Appleton & Co. Chicago: W. T. Keener. Pp. xvii and 872. 1888.

In the eight years which have elapsed since the third edition of Flint's Physiology was issued, such changes have occurred that the author decided to re-write the book rather than to attempt to so remodel the preceding edition as to make it in keeping with the requirements of a text-book on physiology at the present time. Acceptable as the earlier editions were in their day, this fourth edition will be found more so, and the author and publishers alike are to be congratulated upon the results of the work in this edition. The text is clear and concise ; the illustrations are clean cut, and serve to illustrate and explain, not to mislead and confuse, as too often happens in inferior illustrations.

Printed on good paper, with perfect type, makes reading of the book a pleasure.

It is divided into twenty-six chapters, which cover the field of physiology very fully. An effort has been made to present matters now generally accepted as facts, rather than to state conflicting theories yet sub judice. It may, therefore, be accepted as representing the most advanced ideas of human physiology of the present day, and it will continue to be regarded as a standard text-book on the subject. A more detailed review of a work whose general characteristics are so well known would seem superfluous. It merits the commendation it will receive.

A MANUAL OF OPHTHALMIC PRACTICE. By CHARLES HIGGENS, F. R. C. S. E., Lecturer on Ophthalmology at Guy's Hospital Medical School, etc., etc. With illustrations. Philadelphia: P. Blakiston, Son & Co. Chicago: W. T. Keener. Pp. viii and 314. 1888.

This manual is divided into two parts. The *first* consists of twelve chapters, beginning with refraction, followed by the methods of examining the eye, and then considers very briefly affections of the eye and its appendages.

Part *second* consists of seven chapters, and relates to operations, both upon the appendages of the eye and upon the eyeball, with such details relating to operations as will be found serviceable to beginners in ophthalmic work, for whose benefit the work seems chiefly to have been prepared. The author's aim has been to be concise and practical.

HAND-BOOK OF HISTORICAL AND GEOGRAPHICAL PHTHISIOLOGY. By GEORGE A. EVANS, M. D. New York: D. Appleton & Co. Chicago: A. C. McClurg & Co. 1888. 8vo. Pp. v and 295.

This is a brief compilation from all available sources on the history and geographical distribution of tuberculosis of the lung. The author seems to have chosen a most unfortunate and unscientific title. Phthisis should not be used except in its strict etymological sense. It should be forever divorced from any essential relation with pulmonary tuberculosis. The condensation from the "Tenth U. S. Ceusus Report" is a valuable epitome, but it is that alone. The book is written without an argument or object. It overflows with quotations. The most valuable part is the statistical tables showing the death-rate from consumption in each of the States by counties. B. H.

THE MEDICAL NEWS VISITING LIST FOR 1889. Weekly (dated, for 30 patients). Monthly (undated, for 120 patients per month). Perpetual (undated). Philadelphia: Lea Brothers & Co., 706 and 708 Sansom St.

Each in one pocket-size volume, containing 48 pages of indispensable data, with 5 illustrations, and 176 pages of classified blanks, ruled on fine writing paper. Flexible red leather, flap and pocket, pencil, rubber, and catheter-scale, $1.25. Thumb-letter index, 25 cents extra.

Thoroughly revised and brought up to date in every respect. The text portion (48 pages) contains data useful in the daily work of the physician and surgeon, including the latest therapeutic novelties, their doses and effects. The classified blanks (176 pages of paper suitable for pen or pencil) afford space most conveniently arranged for all records of practice, business, etc.

DOCTOR AND PATIENT. By S. WEIR MITCHELL, M. D., LL. D. Philadelphia: J. B. Lippincott Company. Pp. 177. 1888.

The author, in his introductory, says the essays—seven in number—which compose the volume, "deal chiefly with a variety of subjects to which every physician must have given more or less thought. Some of them touch on matters concerning the mutual relations of physician and patient, but are meant to interest and instruct the laity rather than the medical attendant. The larger number have from their nature a closer relation to the needs of women than of men."

Much of the book is a natural outgrowth of the author's experience with women having real or imaginary nervous troubles, and the peculiarities which they exhibit under such circumstances. What is said regarding *The Physician* is a pen-picture of much that is experienced in the everyday life of most physicians.

Regarding his advocacy of camp-life for women there will probably be found wide difference of opinion in the profession and out of it, but he partly forestalls argument by saying "All kinds of modifications of the life I advise are possible."

The essays are written in any easy—almost colloquial—style, leaving an impression that they may have been written as much for the amusement of the writer as of the reader.

